# A Phase II Random, Double-Blind, Placebo-Controlled Study of the Safety and Immunogenicity of a Recombinant G Protein-Based Respiratory Syncytial Virus Vaccine in Healthy Older Adults

**DOI:** 10.3390/vaccines13080885

**Published:** 2025-08-21

**Authors:** Lunan Zhang, Gan Zhao, Xin Cheng, Shuo Wang, Jiarong Wang, Xuefen Huai, Yu Xia, Yanling Xiao, Sulin Ren, Shijie Zhang, Qiao Wang, Bin Wang

**Affiliations:** 1 Key Laboratory of Medical Molecular Virology (MOE/NHC/CAMS), Shanghai Institute of Infectious Disease and Biosecurity, School of Basic Medical Sciences, Fudan University, Shanghai 200032, Chinawangqiao@fudan.edu.cn (Q.W.); 2 Advaccine Biopharmaceuticals Suzhou Co., Ltd., Suzhou 215000, China; 3 Children’s Hospital, Fudan University, Shanghai 200032, China

**Keywords:** respiratory syncytial virus, safety, immunogenicity, vaccine

## Abstract

**Background**: Respiratory syncytial virus (RSV) poses a significant global health threat, particularly to children and the elderly. While progress has been made in RSV vaccine development, gaps remain, especially in protecting the elderly population. BARS13, a recombinant non-glycosylated G protein-based RSV vaccine, has shown promise in preclinical and Phase 1 studies. This phase II trial sought to determine whether escalating doses of BARS13 could enhance immune responses while maintaining safety and tolerability in healthy older adults aged 60–80 years. **Methods**: This study employed a rigorous randomized, double-blind, placebo-controlled design involving 125 participants across two Australian centers. Participants were randomized in a 3:1 (vaccine–placebo) ratio for Cohorts 1–2 (30 active, 10 placebo each) and a 2:1 ratio for Cohort 3 (30 active, 15 placebo). Cohort 1 (low dose) received 10 µg rRSV-G + 10 µg CsA in one arm + a placebo in the other (Days 1 and 29); Cohort 2 (high dose) received 10 µg rRSV-G + 10 µg CsA in both arms (20 µg total per dose, Days 1 and 29); Cohort 3 (multi-dose) received the same dose as that of Cohort 2 but with a third dose on Day 57. The placebo groups received IM injections in both arms at matching timepoints. The primary endpoints included safety and tolerability assessments, while the secondary endpoints evaluated the RSV G protein-specific IgG antibody concentrations using enzyme-linked immunosorbent assays (ELISAs). Statistical analysis was performed on both the safety and immunogenicity populations. **Results**: BARS13 was well-tolerated across all cohorts, with no serious adverse events (SAEs) related to the vaccine. The most common adverse events were mild local reactions (pain and tenderness) and systemic reactions (headache and fatigue), which resolved within 24–48 h. Immunogenicity analysis demonstrated a dose-dependent increase in the RSV G protein-specific IgG geometric mean concentrations (GMCs). Cohort 3, which received multiple high-repeat dose administrations, showed the highest immune response, with the IgG GMC rising from 1195.4 IU/mL on Day 1 to 1681.5 IU/mL on Day 113. Response rates were also the highest in Cohort 3, with 86.2% of participants showing an increase in antibody levels by Day 29. **Conclusions**: BARS13 demonstrated a favorable safety profile and strong immunogenicity in elderly participants, with a clear dose-dependent antibody response. These results support further development of BARS13 as a potential RSV vaccine candidate for the elderly. Further studies are needed to evaluate the long-term efficacy and optimal dosing schedule.

## 1. Background

Respiratory syncytial virus (RSV) remains a major global health burden, posing a considerable threat to the health of children and the elderly. According to a meta-analysis [1], RSV infection is extremely prevalent among children, accounting for 70% of all pediatric respiratory infections, and annually causing approximately 33 million cases of acute lower respiratory tract infections (LTRIs) in children aged <5 years worldwide, which cause an estimated 3.6 million hospitalizations due to RSV and approximately 26,300 deaths attributed to RSV-related hospitalizations. Concurrently, immunosenescence profoundly affects the immune systems of elderly individuals, in which antibody responses are less numerous and efficient compared to those of younger individuals, and dysregulation of cytokine production along with lymph node fibrosis, all of which increase their susceptibility to infectious diseases like RSV. For the elderly population, there were about 5.2 million cases of RSV-related acute respiratory infections (ARIs) among adults aged 60 and above worldwide, with approximately 470,000 hospitalizations, and 33,000 of these hospitalizations ended in death [2]. In high-income countries, it is expected that by 2025, the number of RSV-ARI cases among individuals aged 65 and older could reach 10 million, with 800,000 requiring hospital treatment and 74,000 potentially leading to death in the hospital [3].

The journey of RSV vaccine development has been filled with challenges and innovations. In the early 1960s, an attempt to develop a formalin-inactivated RSV (FI-RSV) vaccine was thwarted due to vaccine-enhanced disease (VED), which served as a cautionary tale for subsequent research [4,5,6]. Since then, scientists have explored a variety of vaccine strategies to prevent severe disease caused by RSV. Advances in structural biology have revealed the potential of stable prefusion F protein subunit vaccines, a discovery that has injected new vitality into RSV vaccine development. Preclinical and clinical studies have shown that vaccine designs targeting the prefusion conformation of the RSV fusion (F) protein elicit stronger neutralizing antibody responses than the postfusion form [7,8].

Currently, the development of RSV vaccines is rapidly advancing. Several RSV candidate vaccines show good efficacy and safety in preventing RSV-related acute respiratory infections [9]. For instance, the RSV prefusion F vaccine has demonstrated 68% efficacy in preventing RSV-related acute respiratory infections, 70% efficacy in preventing RSV-related lower respiratory infections that require medical intervention, and 87% efficacy in preventing severe RSV-related lower respiratory infections in elderly populations.

Despite the progress made in RSV vaccine development, there are still research gaps that need to be filled. The current research on the efficacy and safety of RSV vaccines in different populations is insufficient, particularly among infants [10]. Moreover, further research is needed to assess the long-term protective effects and cost-effectiveness of RSV vaccines. Since the current RSV vaccines and antibodies are mainly based on the RSV prefusion protein (preF), researchers are also exploring the potential of other RSV non-fusion antigens as vaccine immunogens, such as glycoprotein G. This G protein serves as an attachment factor during RSV infection, interacting with receptors on target cells to facilitate viral entry and initiate infection [11]. By developing subunit vaccines targeting the RSV G protein, researchers aim to elicit an immune response that neutralizes the virus before it can enter cells, thereby preventing infection. Promising results from preclinical studies have positioned the G protein as a viable candidate for subunit RSV vaccine development [12,13,14].

Moreover, the use of a bacterially expressed non-glycosylated G protein-based vaccine candidate has demonstrated cross-protective efficacy against both homologous and heterologous RSV challenges in preclinical models, indicating its potential as a broadly protective vaccine candidate [15].

The development of BARS13, a recombinant RSV G protein-based vaccine, represents a strategic approach to addressing key gaps in RSV vaccine research. Unlike other vaccines that focus on the F protein, BARS13 leverages the non-glycosylated G protein, a critical attachment factor in RSV infection, which plays a pivotal role in the virus’s ability to enter host cells. The RSV attachment (G) glycoprotein is a 298-amino acid surface protein heavily glycosylated with up to 35 N-linked glycans, which mask most linear epitopes and limit antibody recognition [14]. Unlike the prefusion F protein, which is the target of licensed RSV vaccines, the G protein is structurally unique and lacks a well-defined neutralizing domain. However, a conserved region known as the “cysteine noose” has been implicated in receptor binding and can elicit neutralizing antibodies. The recombinant G protein used in BARS13 is non-glycosylated and expressed in E. coli, preserving the cysteine noose structure and avoiding glycan-mediated immune evasion. By targeting the G protein, BARS13 offers a novel mechanism of action aimed at neutralizing the virus at the point of entry, potentially offering broader protection against both homologous and heterologous RSV strains [16].

The innovation of using a non-glycosylated form of the G protein with its low dose of CsA lies in its capacity to induce a strong antibody response while avoiding the complexities associated with vaccine-enhanced disease (VED) seen in the early RSV vaccines [4,5]. This non-glycosylated form improves the stability of the antigen and simplifies the manufacturing process, offering cost-effective scalability for widespread vaccine distribution. CsA was included in the BARS13 formulation at a sub-immunosuppressive dose (1000× lower than clinical immunosuppressive dosing) based on prior preclinical studies [15]. In this context, CsA acts as an immunomodulator, enhancing humoral immunity by promoting regulatory T cell (Treg) responses, which mitigate vaccine-enhanced disease (VED) while preserving antigen-specific antibody production. Preclinical studies have demonstrated that this approach not only enhances immunogenicity but also avoids the immune evasion mechanisms often facilitated by the glycosylated forms of viral proteins [14], making BARS13 a promising candidate for durable protection.

Moreover, BARS13 fills a critical gap in vaccine development for older adults, a population historically underserved by the existing RSV vaccine efforts. The elderly are disproportionately affected by RSV due to age-related immune decline, and the effectiveness of the existing vaccines is often limited by pre-existing RSV immunity and weaker vaccine-induced responses in this demographic [17]. By focusing on the G protein, BARS13 has the potential to overcome these challenges, as initial studies have shown it induces a robust humoral response even in individuals with prior RSV exposure.

Recently, we conducted the first Phase 1 clinical study to preliminarily evaluate the safety and immune response of a non-glycosylated G protein plus the immunomodulator CsA as an RSV vaccine in healthy adults aged 18 to 45 [18]. The results showed that no serious adverse events occurred in volunteers after vaccination at different dosage levels. Concurrently, the vaccine induced a strong humoral immune response, with an increase in RSV-specific antibody concentrations [18]. As a promising candidate for the development of a subunit RSV vaccine, we have further conducted phase II clinical studies in elderly populations to assess its safety and immune response.

## 2. Methods

### 2.1. Study Design and Procedures

In this phase II clinical trial (NCT04681833), a randomized, double-blind, placebo-controlled, dose-ranging study was implemented to evaluate the safety, tolerability, and immunogenicity of the respiratory syncytial virus (RSV) vaccine candidate BARS13, which contained purified RSV G recombinant protein and a formulated Cyclosporine A (CsA) sterile solution. This study targeted healthy elderly participants, aged between 60 and 80 years.

Participants were randomly assigned to receive the BARS13 vaccine or placebo, with Cohort 1 consisting of 40 participants and Cohort 2 also comprising 40 participants, both at a 3:1 ratio of vaccine to placebo. Cohort 3 included 45 participants at a 2:1 ratio. Injections were administered on Days 1 and 29 to all cohorts, with Cohort 3 receiving an additional dose on Day 57. The single dosage for Cohort 1 was 10 μg of rRSV G protein plus 10 μg of CsA, whereas Cohorts 2 and 3 received 20 μg of each component.

The trial, conducted from May 2021 to January 2024 across two Australian sites, included three dosing cohorts with a total of 125 participants. Cohorts 1 and 2 received two injections on Days 1 and 29, while Cohort 3 received additional injections on Day 57. This study was approved by the Human Research Ethics Committee (HREC), adhering to the Declaration of Helsinki and ICH GCP guidelines, and informed consent was obtained from participants prior to their involvement. A Safety Review Committee monitored participant safety throughout the trial.

The primary endpoints of this study were focused on the safety and tolerability of BARS13, monitoring the incidence and severity of local and systemic adverse events post-vaccination. The secondary endpoints involved measuring the levels of IgG antibodies specific for the RSV G protein using an enzyme-linked immunosorbent assay (ELISA), offering insights into the vaccine’s immunogenicity.

### 2.2. Participants

In this phase II clinical trial, healthy elderly male and female participants aged between 60 and 80 years were enrolled. Individuals with stable chronic diseases were eligible for inclusion, but those with a history of severe allergies, immunosuppressive therapy, known HIV, HBV, or HCV infections, or those who had recently received other vaccines (except for influenza and SARS-CoV-2 [COVID-19] vaccines, which should not be administered within a 14-day interval of the study vaccine) or had previously received an investigational RSV vaccine were excluded. All participants were required to have a BMI not exceeding 40 kg/m^2^, normal ECG parameters, and stable blood pressure.

### 2.3. The Vaccine

The lyophilized powder of rRSV G protein (14 µg/vial) and CsA diluent (18 µg/0.9 mL/vial) was produced by Advaccine Biopharmaceuticals Suzhou Co., Ltd., located in Suzhou, China. The placebo was a formulation buffer (0.9 mL/vial) without active components. The rRSV G protein lyophilized powder was reconstituted with 0.7 mL of sterile CsA diluent solution as the active BARS13 vaccine and intramuscularly injected with 0.5 mL of reconstituted BARS13 vaccine per arm. The reconstituted vaccine remains stable for ≤4 h at 2–8 °C before intramuscular (IM) deltoid injection.

### 2.4. Safety Assessment

The safety endpoints in this study were designed to evaluate the vaccine’s safety profile, with a particular emphasis on the frequency and intensity of adverse events (AEs) associated with the vaccine. This involved monitoring both solicited AEs, such as local reactions like pain, tenderness, and erythema, as well as systemic reactions like fatigue and myalgia. In addition, unsolicited AEs were also captured for a period extending beyond the initial 7 days. The occurrence of AEs leading to withdrawal, serious adverse events (SAEs), clinically significant laboratory abnormalities, and treatment-emergent, clinically significant changes in vital signs and physical examinations were also systematically tracked. The AE Severity Grading Definitions were as follows: Grade 1 (mild): symptom is noticeable but easily tolerated; Grade 2 (moderate): discomfort sufficient to interfere with normal activities; Grade 3 (severe): incapacitating, prevents work or daily activities; Grade 4 (life-threatening): immediate risk of death, requires urgent intervention.

### 2.5. Immunogenicity Assessment

The immunogenicity assessment was conducted to evaluate the immune response to the vaccinations. Serum samples from participants were collected at predefined timepoints and analyzed for antibodies against the recombinant non-glycosylated G protein (IgG against rRSV G protein) using validated ELISA protocols. For Cohorts 1 and 2, samples were collected on Days 1, 29, 57, and 85, while Cohort 3 provided additional samples on Day 113. The assay was performed by a validated method, with results presented as geometric mean concentration values and the proportion of participants showing an antibody concentration increase post-vaccination.

A quantitative enzyme-linked immunosorbent assay (ELISA) was used to assess the concentrations of G protein-specific IgG antibodies. Plates were coated with rRSV protein G before being blocked. A standard RSV IgG serum (NIBSC, London, UK, Cat No.: 16/284) was serially diluted to set as the standard curve. Human serum samples were diluted (1 in 2000) and placed on the plate for a 1 h incubation. Following washing, goat anti-human IgG (H + L) peroxidase-labeled IgG antibodies (Invitrogen, Carlsbad, CA, USA, Catalog No.: 31410) were added to the plate for a further 1 h incubation, followed by additional washes. TMB (Sigma-Aldrich, St. Louis, MO, USA, Catalog No.: T0440) and a stop solution were used to create a colorimetric signal. An ELISA plate reader (SpectraMax VersaMax, Molecular Devices, Sunnyvale, CA, USA) was used to read the signal. The signal produced was proportional to the amount of analyte present and interpolated from the calibration curve presented on each plate. The concentrations of anti-G protein IgG antibodies in the samples were calculated from the calibration curves (4 PL curve fitting with 1/Y weighting factor).

### 2.6. Statistical Analysis

The safety population and the immunogenicity population were statistically separated. The safety population included all participants who received the investigational treatment, and the data were used to describe the demographic characteristics, baseline features, and safety/tolerability endpoints. The immunogenicity population comprised participants who received any dose of the treatment and completed at least one post-baseline immunological assessment, with data used primarily for immunogenicity endpoints.

The descriptive statistical methods were used to summarize the demographic variables and participants’ adherence and exposure to the study vaccine, presenting these by treatment group. Adverse events were categorized by using MedDRA coding (v24.0), and a comprehensive summary was compiled, including severity and causality assessments, as well as the incidence and severity of injection site reactions.

For the immunogenicity, the RSV G protein-specific IgG antibody concentrations were obtained with the calculated geometric mean concentrations (GMCs) and tracking changes over time. The response criteria and calculated response rates were defined at each timepoint.

## 3. Results

### 3.1. Study Participants

**Figure 1:**
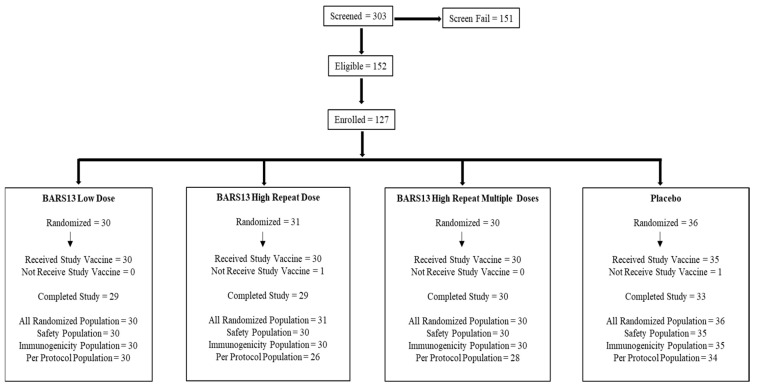
Flow of participants. A total of 303 individuals were screened during the initial phase, with 152 (50.2% of the total) qualifying for this study. These eligible participants were then enrolled and randomly assigned to three distinct dosing cohorts, comprising a total of 125 individuals. The first and second cohorts received two injections on Days 1 and 29, respectively, while the third cohort received an additional injection on Day 57. Allocation of participants was such that the first and second cohorts each contained 40 participants with a 3:1 ratio of vaccine to placebo, indicating that 30 individuals in each received the BARS13 vaccine and 10 received the placebo. The third cohort included 45 participants with a 2:1 ratio, resulting in 30 individuals receiving the vaccine and 15 receiving the placebo. BARS13 (low dose): an amount of 10 µg rRSV-G + 10 µg CsA in one arm + the placebo in the other (Days 1 and 29). BARS13 (high dose): an amount of 10 µg rRSV-G + 10 µg CsA in both arms (20 µg total per dose, Days 1 and 29). BARS13 (multiple high-repeat doses): an amount of 10 µg rRSV-G + 10 µg CsA in both arms (20 µg total per dose, Days 1, 29, and 57).

Over the study period extending from 19 May 2021 to 19 December 2022, a total of 303 individuals underwent screening. Of these, 152 individuals (50.2%) were determined to be eligible for this study. Following enrollment, 127 participants were randomly assigned to receive either the RSV vaccine candidate BARS13 or a placebo (Figure 1). Among the enrolled participants, 125 were identified as the allocated cohorts, with 121 (96.3%) successfully completing this study. The reasons for the non-completion of this study by the remaining participants were multifaceted, encompassing individual discomfort, challenges with venous access, personal circumstances, lifestyle alterations, adverse events, and the withdrawal of consent.

The demographic profile of the safety population (Table 1) was consistent across the dosing groups. The median age was 64.0 years (range: 60 to 77), and the mean BMI was 28.422 kg/m^2^ (SD: 4.6031), with no significant variance observed between the cohorts. There was a near equal distribution of males (63) and females (62). The majority of participants were White (96.8%), with small percentages identifying as Asian (2.4%), multiple-race (0.8%), and Hispanic or Latino (2.4%).

### 3.2. Vaccine Safety and Tolerability

**Figure 2:**
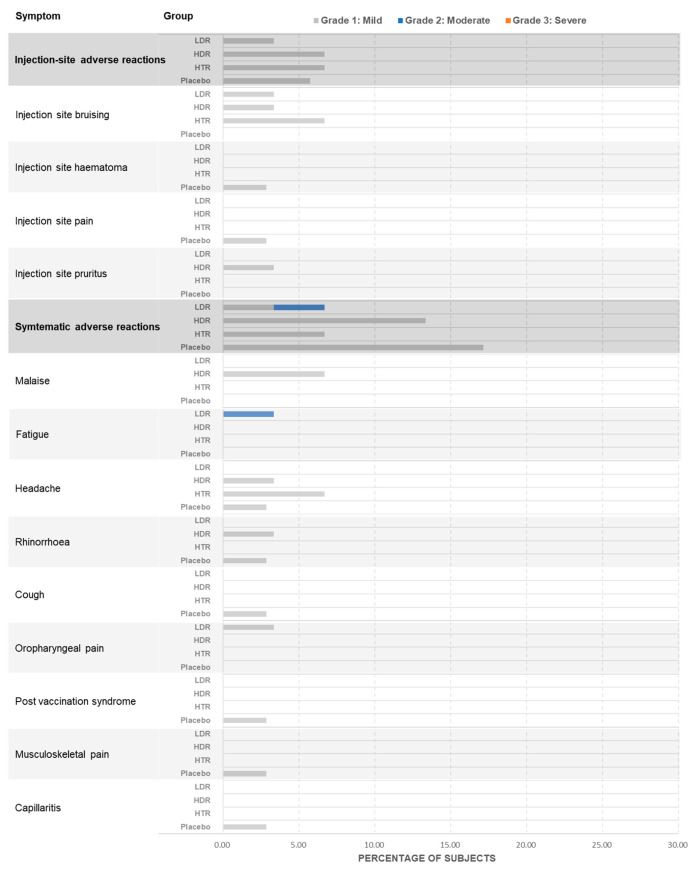
Solicited injection site and systemic adverse events.

No severe (Grade 3 or higher) solicited local reactions were observed. Participants who received the BARS13 low dose or placebo did not experience any moderate (Grade 2) local reactions (Figure 2). On Day 1, all local reactions were mild (Grade 1), except for one case of pain and two instances of tenderness in a participant who received the BARS13 high-repeat dose. On Day 29, the majority of local reactions remained mild, with the exception of one pain event and one tenderness event each in two participants receiving the BARS13 high-repeat dose and one moderate pain event in a participant receiving the BARS13 multiple high-repeat doses. By Day 57, all local reactions reported were mild (Grade 1). Occurrences of bruising, itching, paresthesia, swelling, and erythema were rare and typically resolved within 48 h following vaccination. Solicited systemic reactions were predominantly mild, with headaches being the most prevalent, reaching a peak of 14 cases on Day 1 and 12 on Day 29, and lasting from 1 to 4 h. Fatigue was reported in seven instances on Day 1 and in six on Day 29, with a median duration of 1 to 15 h. Other less common adverse events included arthralgia, diarrhea, dizziness, fever, lightheadedness, malaise, myalgia, and nausea/vomiting, all of which resolved within a median time of 24 h post-vaccination.

No concerning safety findings were reported, and across the study there were 11 serious unsolicited TEAEs reported in 10 participants which occurred in participants administered all dosing regimens of the BARS13 and placebo (low dose: 3 participants with 3 SAEs; high-repeat dose: 2 participants with 2 SAEs; multiple high-repeat doses: 1 participant with 2 SAEs; and placebo: 4 participants with 4 SAEs). There were no serious unsolicited TEAEs that were deemed to be possibly, probably, or definitely related to the study treatment by the principal investigator (PI). Three unsolicited TEAEs led to withdrawal or discontinuation, and these were also deemed unrelated to the study vaccine.

Abnormal clinical hematology parameters were reported in seven participants administered BARS13, which were transient and not dose-related. Most were cases of neutropenia, with the PI considering most of these possibly related to the study treatment. No clinical chemistry or urinalysis parameters were found to be related to the study vaccine, with the exception of a single case of elevated lipase. This instance was considered possibly related to the vaccine and was characterized as mild (Grade 1) in severity, occurring in a participant who had received multiple high-repeat doses of the vaccine.

No vital sign abnormalities were considered clinically significant (CS) by the PI, except for one case of hypertension deemed unrelated to the study vaccine.

### 3.3. Humoral Response to BARS13

Serum samples were collected from participants at predefined timepoints to assess the antibody concentration against the recombinant G protein. For Cohorts 1 and 2, samples were collected on Days 1, 29, 57, and 85, while Cohort 3 provided additional samples on Day 113.

**Figure 3:**
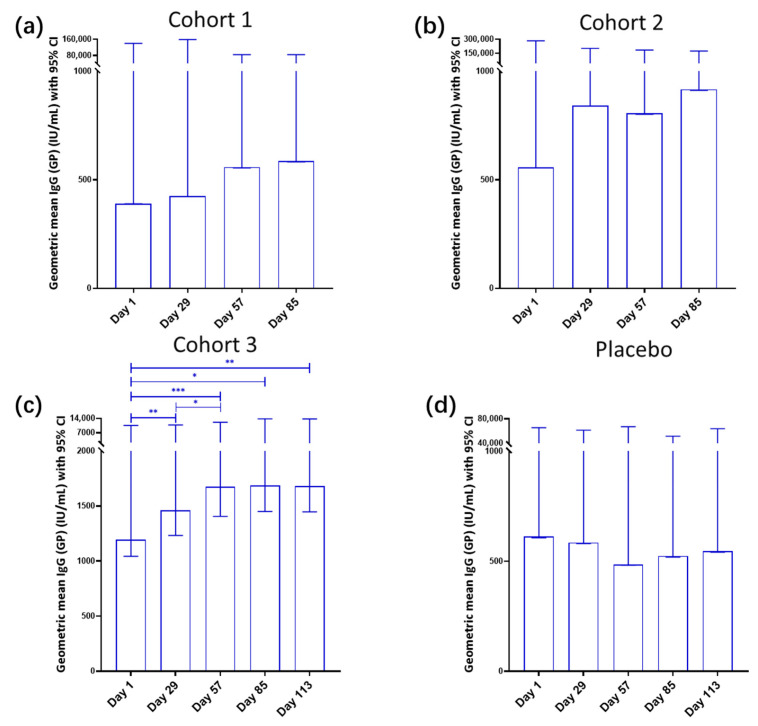
Geometric mean IgG (GP) concentrations over time. (**a**) Cohort 1 (low dose): Participants in Cohort 1 demonstrated a consistent increase in their IgG geometric mean concentration (GMC), starting from 390.9 IU/mL on Day 1 and reaching 586.7 IU/mL by Day 85. (**b**) Cohort 2 (high-repeat dose): This group also observed an upward trend in their IgG GMC, with levels increasing from 557.1 IU/mL on Day 1 to a peak concentration of 917.5 IU/mL on Day 85. (**c**) Cohort 3 (multiple high-repeat doses): Participants in this cohort experienced a marked and enduring elevation in their IgG GMC, beginning at 1195.4 IU/mL on Day 1 and climbing to 1681.5 IU/mL by Day 113. (**d**) Placebo: Recipients of the placebo showed no significant variations, with a geometric mean fold increase (GMFI) from baseline of 1.0 at Day 29, which slightly dipped to 0.9 by Day 57 and then remained stable. Significances are indicated as: * *p* < 0.05; ** *p* < 0.01; *** *p* < 0.001.

Immunogenicity results showed a dose-dependent increase in the IgG geometric mean concentration (GMC) across cohorts, with Cohort 1 (low dose) showing moderate antibody elevation from 390.9 IU/mL on Day 1 to 586.7 IU/mL on Day 85, Cohort 2 (high-repeat dose) demonstrating stronger responses from 557.1 IU/mL on Day 1 to a peak of 917.5 IU/mL on Day 85, and Cohort 3 (multiple high-repeat doses) achieving the highest sustained levels from 1195.4 IU/mL on Day 1 to 1681.5 IU/mL on Day 113. Placebo recipients displayed no significant changes, with a geometric mean fold increase (GMFI) from baseline of 1.0 at Day 29, slightly decreasing to 0.9 by Day 57, and remaining stable thereafter (Figure 3).

Overall, this study indicated a dose-dependent increase in the IgG (GP) GMC among BARS13 recipients, with the most pronounced response in the group receiving multiple high doses.

Individual participant response rates, defined as the percentage increase in antibody levels from the pre-vaccination baseline, were higher in BARS13 recipients across all dosages compared to placebo recipients. Peak response rates were achieved by Day 29 in all BARS13 groups, with a minor decline by the final follow-up. Notably, the multiple-high-repeat-dosage group sustained a robust response rate of 76.7%. The low-dosage group maintained a consistent 63% response rate, while the high-repeat-dosage group saw rates peak at 82.8% on Day 29, dip to 64.3% by the third follow-up, and recover to 76.7% by the fourth. The multiple-high-repeat-dosage group exhibited an initial response rate of 86.2% on Day 29, which settled at 76.7% by the fourth follow-up. Placebo recipients, in contrast, exhibited markedly lower response rates throughout (Table 2).

These findings underscore that BARS13 led to a significant and dose-dependent enhancement of the antibody response against RSV in all cohorts. Although minor fluctuations in the response rates were noted, the sustained high levels across the BARS13 dosages reflect the vaccine’s ability to trigger an immune response in the majority of participants. Further research is necessary to refine the vaccination approaches and assess the longevity of immune responses.

## 4. Discussion

This phase II study evaluates the safety, tolerability, and immunogenicity of BARS13 in healthy elderly males and females aged 60 to 80. Consistent with prior Phase I findings, this phase II trial confirms the favorable safety profile and strong immunogenicity of BARS13.

The findings demonstrate that the BARS13 vaccine is well-tolerated among elderly participants, with no serious adverse events reported across the dosage groups. Participants in all dosage groups demonstrated good tolerance to the vaccine, with no serious adverse events (SAEs) observed. All treatment-emergent adverse events (TEAEs) were mild to moderate and not clearly related to vaccination. These TEAEs mainly included local reactions such as pain and tenderness, as well as systemic reactions such as headache and fatigue, which were usually transient and resolved within 24 to 48 h. Furthermore, no clinically significant vital sign changes related to vaccination were recorded during this study, providing additional assurance for the safety of BARS13.

Immunogenicity assessments revealed that after vaccination with BARS13, there was a significant increase in the levels of IgG antibodies against the RSV G protein in the participants. This increase was dose-dependent, meaning that as the vaccine dose increased, the observed immune response was stronger. In the low-dose group (Cohort 1), the geometric mean concentration (GMC) of IgG steadily rose from before vaccination to Day 85, indicating that the vaccine successfully induced an immune response. In the high-dose groups (Cohort 2 and Cohort 3), the observed increase in the IgG GMC was even more pronounced, indicating that increasing the vaccine dose and frequency of administration can enhance the immune response.

The elderly population is frequently susceptible to respiratory syncytial virus (RSV) infections, which can pose a challenge to the efficacy of RSV vaccines [3]. This is mostly attributed to the presence of pre-existing antibodies that neutralize the vaccine antigens and, hence, impede the vaccination’s effectiveness. Developing an effective respiratory syncytial virus (RSV) vaccination for this particular age range presents a significant hurdle. Furthermore, pre-existing medical disorders and the concurrent use of medications may exacerbate the challenges associated with the effectiveness of the vaccine in older populations. Additionally, the immune systems of older individuals tend to weaken over time, making it more difficult for vaccines to generate a strong and lasting immune response. This further emphasizes the need for the careful evaluation and consideration of the dosing escalation and frequency to ensure the optimal effectiveness of an RSV vaccine in older populations. An analysis of the anti-RSV-G IgG antibody baseline revealed that subjects with a history of environmental exposure to RSV infections exhibited a significantly elevated baseline level of this antibody. To eliminate inconsistency among individuals, we measured the quantities of anti-G IgG. These concentrations were reported in international units per microliter (IU/mL), along with 95% confidence intervals for each treatment group.

The administration of two doses of low-dose BARS13 (10 µg) via injection induced significant anti-G IgG antibodies at Week 4 after vaccination and more than 60% of the participants exhibited responsiveness. The cohorts administered a high dosage of BARS13 (20 µg) had sustained production of antibodies at both Weeks 4 and 8. Furthermore, these antibodies exhibited an enhanced response towards BARS13. An additional dose of BARS13 led to a substantial boost in antibody production, surpassing baseline levels at every measured timepoint, highlighting the benefit of repeated dosing. This demonstrated a dose-dependent increase pattern from the low-dose to high-dose cohorts, as evidenced by the rise from baseline to post-vaccination. As evidenced by the antibody levels in Cohort 3, which increased further after the second and third doses, the boost dose also played an important role in sustaining high levels in the antibody response.

These results position BARS13 as a competitive candidate among the current RSV vaccines, particularly due to its unique targeting of the G protein rather than the more commonly studied F protein [18]. While many recent efforts have focused on the prefusion F protein, which has been shown to elicit potent neutralizing antibodies, BARS13 targets the G protein, providing a complementary mechanism of action. By preventing viral attachment and entry into host cells, BARS13 addresses a critical phase of RSV infection, potentially offering a broader spectrum of protection. The dose-dependent antibody responses observed in this study highlight BARS13’s potential to induce robust immunity, even in the elderly, a population that has historically shown diminished responses to vaccines targeting the F protein. This distinction may prove advantageous in populations with pre-existing RSV immunity, where the G protein’s immunogenicity may not be as influenced by previous exposure as the F protein is.

Furthermore, the use of the non-glycosylated form of the G protein in BARS13 differentiates it from other RSV vaccines and offers potential benefits in terms of vaccine stability and safety. The avoidance of glycosylation could minimize the risk of vaccine-enhanced disease (VED), a concern that has lingered since the failure of the early RSV vaccines. By circumventing this risk, BARS13 could offer a safer profile in vulnerable populations, particularly the elderly, who are at increased risk for severe RSV infections [19].

A prior clinical study revealed that the importance of dosage was established in a dose-ranging investigation of extracted and purified F, G, and M antigens from RSV A viruses through intramuscular injection at doses of 100, 50, or 25 µg to the elderly population. The antibody levels against RSV F and G proteins were found to be equal when administering doses of 25 and 50 µg of the vaccine. However, a considerably greater antibody response was achieved after administering a dose of 100 µg of the vaccine in comparison to the 25 and 50 µg doses [20]. These findings support the need for dose optimization in elderly populations, where immune senescence may require higher antigen exposure to achieve protective immunity. Further studies are needed to determine the optimal dosage for any protection against RSV. These results are significant, as they indicate that the immune response to RSV can be enhanced by increasing the vaccine dosage. This is particularly important for the elderly population, as they are more susceptible to severe RSV infections. The findings warrant further investigation into the safety and efficacy of higher vaccine doses and could potentially lead to the development of a more effective vaccine against RSV in older adults. Dose selection, as observed in this current study, is pivotal for G protein-based vaccines in the elderly population. The elderly population often has weaker immune responses to vaccines, making it necessary to administer higher dosages to achieve optimal protection against RSV. Additionally, our research suggests that dosing selection is critical in G protein-based vaccines for older adults, as it directly impacts the vaccine’s efficacy and effectiveness. These findings underscore the need for more research and development in this area to improve the immunity and health outcomes of the elderly population [21].

Despite promising findings, there are several limitations that should be acknowledged. First, the immunogenicity follow-up period was relatively short, limiting the ability to assess the long-term durability of the immune response. Future studies should extend the follow-up period to determine whether the antibody responses elicited by BARS13 are sustained over time and provide long-term protection against RSV. Second, the sample size, while sufficient for detecting short-term safety and immunogenicity signals, may limit the generalizability of the findings, particularly regarding rare adverse events or variability in immune responses across more diverse populations. A larger, more ethnically diverse cohort would strengthen the conclusions and provide a clearer understanding of BARS13’s efficacy across different demographics. Moreover, while our ELISA included a standard neutralizing antibody control from the NIBSC, it did not directly measure viral neutralization. We are currently optimizing a primary human airway epithelial cell-based neutralization assay for RSV G-specific antibodies, which we plan to include in future studies. Due to the lack of a validated cell line-based neutralization assay for G protein-based vaccines, we were unable to perform this assay in the current trial.

Additionally, the absence of direct comparisons to other RSV vaccines in this study prevents a full assessment of how BARS13’s safety and immunogenicity profile stacks up against those targeting the prefusion F protein. Future studies could consider head-to-head trials to evaluate the clinical advantages and immunological differences between G protein-based and F protein-based vaccines, particularly in elderly and high-risk populations. Lastly, the cost-effectiveness of BARS13 has yet to be evaluated, and given the global burden of RSV, especially in low- and middle-income countries, future studies should assess whether BARS13 can be produced and distributed at a competitive cost compared to other RSV vaccines.

Overall, this phase II study in older adults showed different dose levels of the recombinant G protein-based investigational RSV vaccine to be safe, well-tolerated, and highly immunogenic and responsive in adults 60–80 years of age. Further research and development in this area is crucial in order to determine the optimal dosage and administration schedule of the recombinant G protein-based investigational RSV vaccine for maximum effectiveness in elderly individuals. Additionally, studying the long-term effects of the vaccine and its potential impact on reducing hospitalizations and mortality rates among older adults will provide valuable insights into improving their overall health outcomes. Ultimately, investing in the continued development and implementation of the recombinant G protein-based RSV vaccine has the potential to significantly enhance the immunity and well-being of the elderly population.

## 5. Conclusions

In summary, this phase II clinic trial has demonstrated that BARS13, a recombinant G protein-based RSV vaccine, not only showed a good safety and tolerability profile across different dose groups in an older population but also, importantly, could induce a meaningful anti-G antibody in a dose- and frequency-dependent manner. The continuous upward trend in the antibody concentration is promising for the effectiveness of BARS13.

## Figures and Tables

**Table 1:** Summary of demographics (safety population).

Variable Statistic	BARS13 Low Dose (N = 30)	BARS13 High-Repeat Dose (N = 30)	BARS13 Multiple High-Repeat Doses (N = 30)	Pooled Placebo (N = 35)	All Participants (N = 125)
Age (Years)					
Mean	65.2	65.6	64.3	65.9	65.3
Std Dev	4.87	4.21	4.07	4.63	4.45
Median	64.0	65.5	62.5	65.0	64.0
Minimum	60	60	60	60	60
Maximum	76	77	74	77	77
Sex (n (%))					
Male	16 (53.3)	12 (40.0)	18 (60.0)	17 (48.6)	63 (50.4)
Female	14 (46.7)	18 (60.0)	12 (40.0)	18 (51.4)	62 (49.6)
Ethnicity (n (%))					
Hispanic or Latino	1 (3.3)	2 (6.7)	-	-	3 (2.4)
Not Hispanic or Latino	28 (93.3)	28 (93.3)	29 (96.7)	34 (97.1)	119 (95.2)
Not Reported	1 (3.3)	-	-	1 (2.9)	2 (1.6)
Unknown	-	-	1 (3.3)	-	1 (0.8)

Notes: 1. Abbreviations: n: number of participants. N: number of participants in group. 2. Age: The average age across all participants was 65.3 years, with the youngest participant being 60 years old and the oldest being 77 years old. 3. Sex Distribution: This study had a nearly equal distribution of male and female participants, with 50.4% being male and 49.6% being female. Cohort 3 had the highest percentage of male participants (60.0%), while Cohort 2 had the lowest (40.0%). 4. Race: The majority of participants identified as White (96.8%). 5. Ethnicity: Most participants were not Hispanic or Latino (95.2%). There were a few participants who identified as Hispanic or Latino, with the highest representation in Cohort 1 (3.3%). 6. Body Mass Index (BMI): The mean BMI across all participants was 28.422 kg/m^2^. The range of BMI values was wide, from a minimum of 18.96 kg/m^2^ to a maximum of 39.89 kg/m^2^.

**Table 2:** Summary of IgG (GP) responses over time (immunogenicity population).

Timepoints		Cohort 1 (N = 30)	Cohort 2 (N = 30)	Cohort 3 (N = 30)	Pooled Placebo (N = 35)
Day 29	n	30	29	29	35
	Responders	19 (63.3%)	24 (82.8%)	25 (86.2%)	12 (34.3%)
	Non-responders	11 (36.7%)	5 (17.2%)	4 (13.8%)	23 (65.7%)
Day 57	n	-	-	28	15
	Responders	-	-	23 (82.1%)	4 (26.7%)
	Non-responders	-	-	5 (17.9%)	11 (73.3%)
Follow-up Visit 3	n	29	28	29	35
	Responders	18 (62.1%)	18 (64.3%)	21 (72.4%)	9 (25.7%)
	Non-responders	11 (37.9%)	10 (35.7%)	8 (27.6%)	26 (74.3%)
Follow-up Visit 4	n	30	30	30	33
	Responders	19 (63.3%)	23 (76.7%)	23 (76.7%)	7 (21.2%)
	Non-responders	11 (36.7%)	7 (23.3%)	7 (23.3%)	26 (78.8%)

Notes: A response was defined as any increase greater than the baseline value in the post-baseline IgG (GP) concentration. IgG: Immunoglobulin G; n: number of participants; N: number of participants in group; GP: RSV G protein.

## Data Availability

The data generated or analyzed during this study are included in this published article.
